# Plasticity and stability in recurrent neural networks

**DOI:** 10.1186/1471-2202-12-S1-P120

**Published:** 2011-07-18

**Authors:** Friedemann Zenke, Guillaume Hennequin, Henning Sprekeler, Tim P Vogels, Wulfram Gerstner

**Affiliations:** 1 School of Computer & Communication Sciences and Brain-Mind Institute, École Polytechnique Fédérale de Lausanne, 1015, Switzerland

## 

Over the past 30 years, Bienenstock-Cooper-Munro (BCM) [1] type learning rules have shaped our understanding of synaptic plasticity. While they excel at explaining the emergence of receptive fields and stimulus selectivity in networks with feed-forward architecture, their impact on recurrent scenarios is less distinctive.

Here, we analyze general BCM-type synaptic plasticity rules with a homeostatic sliding threshold in the framework of recurrent networks of rate-based and spiking neurons. We begin by considering the effects of learning rate and homeostatic timescales on network stability in a non-linear firing rate model. We show how a sensible choice of timescales leads to stable weight dynamics, but other seemingly sensible parameter choices will inevitably lead to catastrophic run-away potentiation. We discuss under which conditions a stable fixed-point in a regime of Hebbian learning exists.

We then study the network's response to perturbations and quantify the critical point whereupon network stability is compromised. By viewing perturbation as a consequence of pattern storage in synaptic connections, we quantify the number of such patterns that can be learned safely in a given time.

Our model could provide simple explanations as to why memory intake capacity is limited and why learning becomes increasingly inefficient during intensive learning periods. We confirm these findings in numerical simulations of spiking neural networks and show that our analytical results apply to synapses subject to additive triplet-STDP [2].
